# Evaluation of Facial Pain with Magnetic Resonance Imaging Neurography of the Trigeminal Nerve

**DOI:** 10.11607/ofph.3470

**Published:** 2023-11-17

**Authors:** Rafael Maffei Loureiro, Daniel Vaccaro Sumi, Hugo Luis de Vasconcelos Chambi Tames, Carolina Ribeiro Soares

**Affiliations:** Department of Radiology and Diagnostic Imaging; Hospital Israelita Albert Einstein

Magnetic resonance imaging (MRI) plays a fundamental role in evaluating patients experiencing facial pain related to trigeminal nerve lesions or diseases. Conventional MRI with volumetric, heavily T2-weighted sequences is commonly employed to visualize the intracranial segments of the trigeminal nerve, aiding in the diagnosis of trigeminal neuralgia (both classical and secondary). However, this approach has limitations in visualizing the extracranial segments of the nerve.^1^ In contrast, MRI neurography (MRIN), an advanced technique in which dedicated high-resolution sequences are used to enhance the visualization of the peripheral nerves, provides detailed assessment of the facial branches of the trigeminal nerve. This nerve-selective imaging technique enables the diagnosis of disorders affecting the extracranial segments of the trigeminal nerve, particularly posttraumatic trigeminal neuropathy.^2,3^

MRIN is considered a safe, noninvasive, radiation-free, and painless procedure, although discomfort from lying still or claustrophobia may occur. A high-field MRI magnet, typically 3-Tesla, is essential to produce neurographic images with high contrast and spatial resolution, enabling multiplanar reformatting for evaluating the trigeminal nerve’s terminal branches. A gadolinium-based contrast agent may be injected intravenously to enhance visualization. Examples of MRIN sequences are provided in Appendix Table 1. Normal nerves typically exhibit intermediate signal intensity and a gradual decrease in caliber along their distal course (Fig 1). Abnormal findings may include increased signal intensity, caliber abnormalities, irregular course, discontinuity, mass lesions, or focal neuroma (Figs 2 and 3).^2,3^

**Fig 1:**
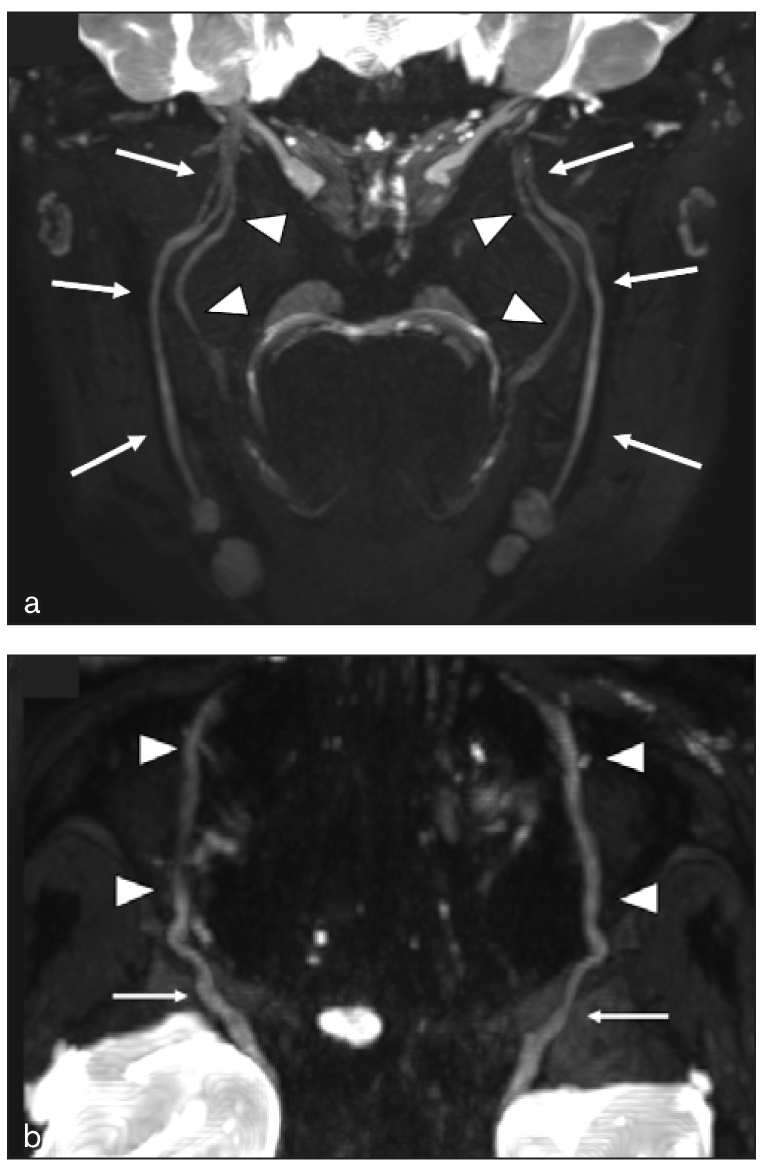
Normal anatomy of the main terminal branches of the trigeminal nerve on MRIN. (a) Oblique coronal contrast-enhanced 3D-STIR shows the inferior alveolar (arrows) and lingual (arrowheads) nerves, which are the main branches of the mandibular nerve (V3). (b) Axial 3D-STIR at the level of the orbital floor shows the infraorbital nerves (arrowheads), which are the main branches of the maxillary nerve (V2) (arrows).

**Fig 2:**
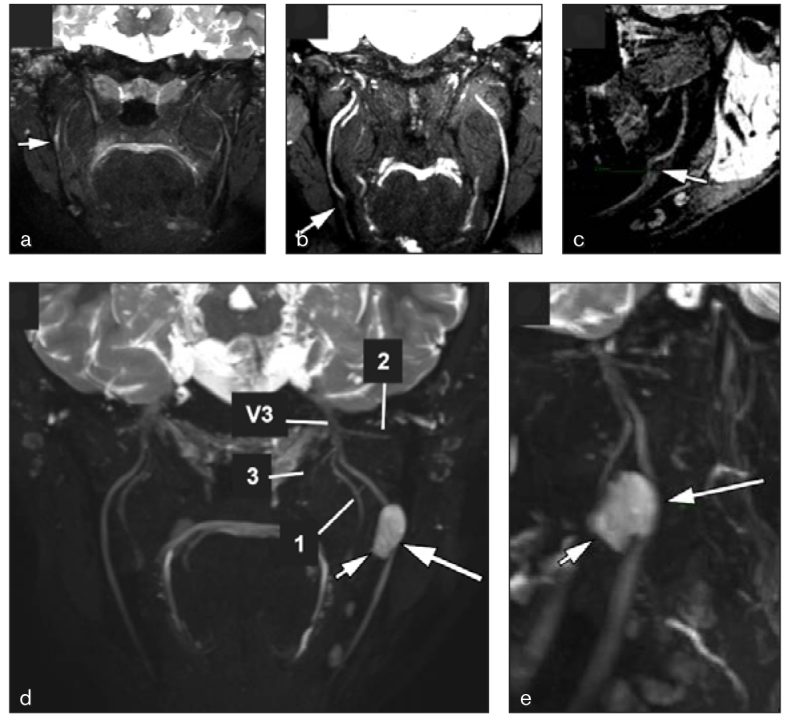
Examples of pathologic cases on MRIN. (a) Oblique coronal contrast-enhanced 3D-STIR image shows segmental thickening and increased signal of the right inferior alveolar nerve (arrow) in a patient with right inferior alveolar neuritis. (a) Oblique coronal and (c) sagittal 3D-PSIF images show a 2-mm discontinuity of the right inferior alveolar nerve (arrow) in a patient with an iatrogenic injury after extraction of the adjacent third molar. (d) Oblique coronal and (e) sagittal contrast-enhanced 3D-STIR images show compression of the left inferior alveolar nerve (long arrows) by an odontogenic keratocyst in the mandibular angle (short arrows). Note the normal anatomy of the left mandibular nerve (V3) and its branches: lingual (1), auriculotemporal (2), and the branch to the medial pterygoid muscle (3).

**Fig 3:**
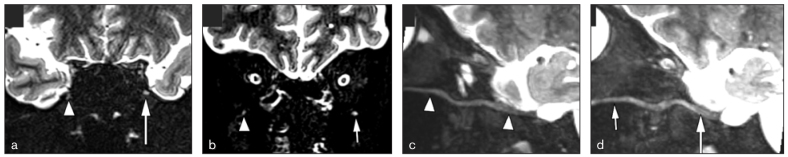
Example of neuritis of the left maxillary and infraorbital nerves. (a and b) Coronal, (c) right sagittal, and (d) left sagittal contrast-enhanced 3D-STIR images show diffuse thickening and increased signal involving the left maxillary nerve at the foramen rotundum (long arrows) and the left infraorbital nerve along the infraorbital canal (images b and d; short arrows). Note the normal appearance of the right maxillary and infraorbital nerves (arrowheads) for comparison.

MRIN plays a crucial role in evaluating posttraumatic neuropathy of the trigeminal nerve’s terminal branches, specifically the inferior alveolar and lingual nerves. These nerves are susceptible to injury during dental procedures, particularly mandibular molar extractions. MRIN has proven to be valuable in detecting and grading nerve injuries, showing a strong correlation between imaging, neurosensory testing, and surgical outcomes.^4,5^ A recent systematic review highlighted the promising potential of MRIN in diagnosing posttraumatic trigeminal neuropathy, determining treatment options, and predicting neurosensory recovery. However, it is important to note that only eight studies met the eligibility criteria for this review. These studies exhibited diverse methodologies and significant heterogeneity in image acquisition, interpretation, and reporting.^5^

In patients experiencing facial pain, MRIN can be a valuable tool to diagnose trigeminal nerve diseases, narrow down potential causes, aid in preoperative planning, and monitor patients after treatment, ultimately enhancing patient care. As the field of MRIN continues to advance, future studies, particularly prospective blinded studies with standardized imaging protocols, will be needed to comprehensively evaluate its impact on clinical practice.^5^

## Appendix

**Appendix Table 1:** Examples of MRIN Sequences

Sequence	Technique	Main parameters	Comments
3D-PSIF	Reversed fast imaging in steady-state free precession	Voxel size: 0.9 mm (isotropic) Acquisition time (min:sec): 7:30	Nerve-selective craniofacial neurography technique.^1^
3D-STIR	Short tau inversion-recovery	Voxel size: 1.5 mm (isotropic) Acquisition time (min:sec): 7:15	Excellent for mandibular nerves. Gadolinium administration is preferable.^1^
3D-CRANI	Contrast-enhanced black-blood short tau inversion-recovery	Voxel size: 0.9 mm (isotropic) Acquisition time (min:sec): 6:45	Gadolinium administration significantly improves fat suppression quality and nerve visualization.^2,3^
